# Detection of OSR2, VAV3, and PPFIA3 Methylation in the Serum of Patients with Gastric Cancer

**DOI:** 10.1155/2016/5780538

**Published:** 2016-04-07

**Authors:** Wen-han Li, Zhang-jian Zhou, Tian-he Huang, Kun Guo, Wei Chen, Ying Wang, Hao Zhang, Yong-chun Song, Dong-min Chang

**Affiliations:** ^1^The First Affiliated Hospital of Xi'an Jiaotong University, Xi'an, Shaanxi, China; ^2^No. 2 Subsidiary Hospital of No. 4 Military Medical University, Xi'an, Shaanxi, China

## Abstract

*Aim*. This study was to evaluate the diagnostic value of OSR2, VAV3, and PPFIA3 hypermethylation in gastric cancer (GC) patients.* Patients and Methods*. By using methylation-specific polymerase chain reaction (MSP), we detected the methylation status in tissue and serum samples from 48 gastric cancer (GC) patients and 25 normal individuals.* Results*. We found that OSR2, VAV3, and PPFIA3 were methylated in 70.8% (34/48), 54.2% (26/48), and 60.4% (29/48) of GC tissue, respectively. On the contrary, those genes were barely methylated in their paired paracancerous histological normal tissues (PCHNTs) (all *P* values < 0.01). We next analyzed the methylated OSR2, VAV3, and PPFIA3 in serum DNA. Compared with 25 normal individuals, those three genes were significantly hypermethylated in GC patients serum samples (all *P* values < 0.01). Regarding their diagnostic value in serum samples, the combined sensitivity of at least one positive among the three markers in serum was 83.3%, with a specificity of 88%.* Conclusion*. Our test suggested that methylation of OSR2, VAV3, and PPFIA3 genes in serum sample may offer a good alternative in a simple, promising, and noninvasive detection of GC.

## 1. Introduction

Although the incidence of gastric cancer (GC) has declined in the past decades, it is still one of the most common malignancies worldwide. According to related data, the annual diagnosis number of GC approximately reaches one million; among them 42% are from China [1, 2]. As GC is usually asymptomatic until advanced stage, it is often associated with poor treatment outcome and low 5-year survival rate. Therefore, an ideal screening tool to detect GC with high sensitivity and specificity has a high priority. Upper endoscopy can distinguish between cancerous and noncancerous conditions by performing biopsies of suspicious areas, therefore being considered as the golden standard for GC detection. However, considering its invasive operations, high-risk patients of GC are reluctant to take this approach as a regular examination. Serum tumor markers, such as carcinoembryonic antigen (CEA), carbohydrate antigen (CA) 19-9, and carbohydrate antigen (CA) 724, have been widely applied in clinical practice. However, none of them are suitable for early detection of GC. Till now, there is no ideal diagnostic method with relatively high sensitivity that could be applied in clinical screening for GC.

As we all know, GC is a multistep process and accumulating data have elucidated that epigenetic alterations, especially DNA methylation, play an important role in GC initiation. By silencing the tumor suppressor genes which play a key role in DNA repair, cell adhesion, cell cycle control, and apoptosis [3], DNA hypermethylation at CpG islands in or near promoter regions contributes a lot during the process of carcinogenesis. Moreover, it has been shown that changes of methylation in body fluids paralleled other somatic tissues and therefore are thought to be connected with certain cancers [4, 5]. On the basis of those studies above, many attempts have been made to investigate biomarkers in serum, urine, and sputum in various malignancies [6–11]. As for GC, many researchers thought serum DNA-based technique is a promising alternative for its relatively noninvasive operations as well as a convenient tissue to assay for constitutional methylation. Many studies have been made to explore the feasibility of the use of serum as a biomarker for certain cancers [4, 5, 12–18].

The selection of the candidate gene for analyzing is crucial to improving the sensitivity and specificity of methylation DNA test. Current microarray technology provides us with an opportunity for high-throughput unbiased methylation analysis of a large number of CpG sites [19]. By using an Infinium HumanMethylation 450 BeadChip array, Zong and colleagues [20] identified three genes (OSR2, VAV3, and PPFIA3) that were hypermethylated in GC tissue. Odd-skipped related 2 (OSR2), which contains DNA-binding C2H2-type zinc finger domains in the C-terminal half, plays an important role in cellular quiescence and proliferation under epigenetic regulation [21, 22]. Additionally, recent articles have reported that VAV3 (a member of VAV gene family which plays an important role in the process of tumor development and metastasis) and PTPRF-Interacting Protein Alpha-3 (PPFIA3) may associated with the tumorigenesis and development of GC [20, 23]. In the present study, we sought to explore the feasibility of DNA methylation status of OSR2, VAV3, and PPFIA3 as a noninvasive screening tool for GC.

## 2. Materials and Methods

### 2.1. Collection of Tissue and Peripheral Blood Samples

In order to reduce bias, we designed this experiment as a blinded assay. All sample collection and preservation were taken care of by a person who did not participate in the follow-up studies. Patients with primary gastric cancer who participated in this study were recruited consecutively from February 2012 to August 2013. The study material consisted of 48 tumor tissue samples, paired paracancerous histological normal tissues (PCHNTs) which are obtained during curative surgery, and the patients' whole blood samples preoperatively. In the meantime, another 25 blood samples from healthy individuals were also obtained. None of the experimental subjects had received prior gastric resection or preoperative chemotherapy/radiation therapy. All samples were immediately frozen and stored at −80°C until DNA was extracted. In order to reduce bias, samples were randomly coded before processing. All patients voluntarily joined this study with written informed consents to have their biologic specimens analyzed. This study was announced by the Ethical Committee of the First Affiliated Hospital of Xi'an Jiaotong University.

### 2.2. DNA Isolation

DNA was extracted from tissues (10 ± 1 mg) with the TIANamp Genomic DNA Kit and for serum samples (400 *μ*L) by use of TIANamp Blood DNA Kit (Tiangen, China). All procedures were strictly carried out according to the manufacturer's instructions. The concentration of DNA was measured by ultraviolet spectrophotography and the quality of DNA was tested by PCR amplification of the human *β*-actin.

### 2.3. Bisulfite Modification

As to bisulfite genomic DNA modification, 2000 Ng of DNA was modified by EpiTect Bisulfite Kit (Qiagen) to convert all unmethylated cytosine to uracil. The bisulfite-treated DNA was eluted in 20 *μ*L of TE buffer and stored at −20°C until being processed.

### 2.4. Methylation-Specific Polymerase Chain Reaction (MSP)

After the bisulfite treatment, we used methylation-specific PCR to testify the methylation status of the OSR2, PPFIA3, and VAV3 promoter. The primers specific to methylated and unmethylated sequences and annealing temperature are summarized in Table 1. We repeated each experiment at least three times in order to reduce false results.

Water without DNA was used as a negative control. Product was visualized by electrophoresis in a 2% agarose gel and analyzed by a gel imaging system. The methylation pattern result was judged by the distribution of visible bands.

### 2.5. CEA, CA19-9, and CA-724 Measurements

Normal levels of CEA, CA19-9, and CA-724 were defined as <3.4 ng/mL, <39 U/mL, and <9.8 U/mL, respectively. The tests were done independently at the clinical laboratory in the First Affiliated Hospital of Xi'an Jiaotong University College of Medicine.

### 2.6. Statistical Analysis

The methylation status of those three genes and all other qualitative variables were expressed as frequencies and percentages (%). The relationship between the methylation status of serum samples and the clinicopathological characteristics was calculated using Fisher's exact test or chi-square test. The correlation of each gene methylation status between GC and their matched gastric nontumorous tissues or serum samples was calculated using Fisher's exact test or chi-square test. Statistical analyses were performed with the SPSS 13.0 software. *P* values < 0.05 (two-sided) were considered statistically significant.

## 3. Results

### 3.1. Patient Characteristics

In order to explore the methylation status of OSR2, VAV3, and PPFIA3 in GC, 48 GC patients (including 39 males and 9 females) and 25 healthy individuals (including 18 males and 7 females) were enrolled in our study. The mean age of GC patients and healthy controls was 56.75 ± 10.6 and 53.48 ± 14.43, respectively. There was no significant difference with respect to age and gender between cases and controls (age: *P* = 0.55; gender: *P* = 0.365).

### 3.2. Gene Promoter Hypermethylation in Tissue Samples

**Figure 1:**
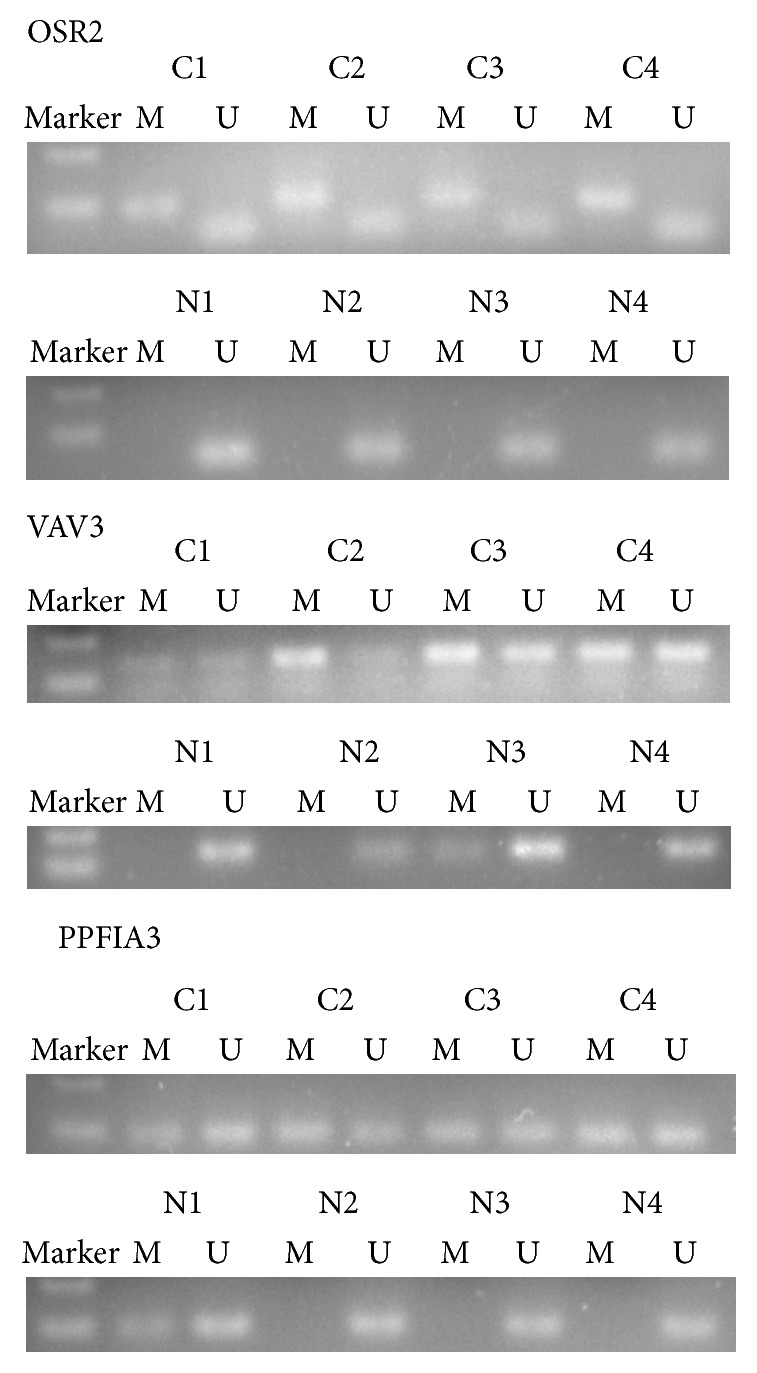
Detection of methylated (M) and unmethylated (U) OSR2, VAV3, and PPFIA3 in tissue of gastric cancer (C1–C4) and paired paracancerous histological normal mucosa (N1–N4).

We first examined the methylation status of OSR2, VAV3, and PPFIA3 in the tissue DNA of GC patients and PCHNTs. The results of representative MSP cases are shown in Figure 1. All of the three genes detected by MSP in GC group showed positive results in unmethylated promoter regions, indicating that there exist some nonneoplastic cells in cancer tissue samples. The prevalence of methylation of these 3 genes was shown in Table 2. Out of 48 GC tissue samples, 34 (70.8%), 26 (54.2%), and 29 (60.4%) exhibited OSR2, VAV3, and PPFIA3 hypermethylation, respectively. On the contrary, in the PCHNTs group, hypermethylation of those three genes was rarely found (OSR2: 4%; VAV3: 0%; PPFIA3: 4%). The data indicated that methylated OSR2, VAV3, and PPFIA3 DNA in tissue were significantly higher in GC patients than those of controls (all *P* values < 0.01).

### 3.3. Gene Promoter Hypermethylation in Serum Samples

**Figure 2:**
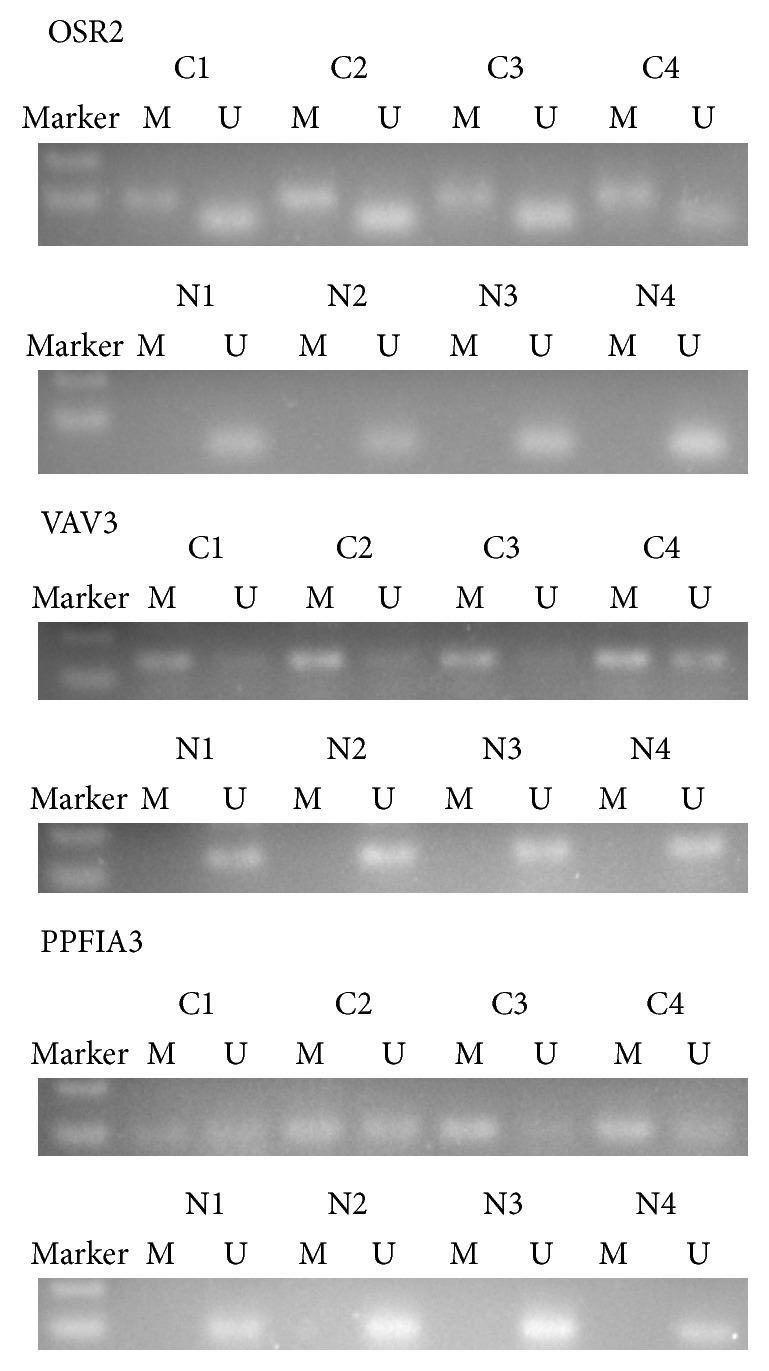
Detection of methylated (M) and unmethylated (U) OSR2, VAV3, and PPFIA3 in serum of gastric cancer (C1–C4) and normal individuals (N1–N4).

To further investigate whether those three genes' methylation could be used as a biomarker for GC, we detected the methylation frequency of those three genes in serum samples between GC patients and healthy controls. Methylation of OSR2, VAV3, and PPFIA3 was detected in 30 (62.5%), 22 (45.8%), and 27 (56.3%) of the serum of 48 GC patients, respectively (Table 2; Figure 2), whereas those genes were weakly methylated in the healthy control group (OSR2: 8%; VAV3: 0%; PPFIA3: 4%). By analyzing the data in tissue samples, we found that all serum samples with aberrant methylation were accompanied with methylation in the corresponding tumor samples. The above evidence suggested that OSR2, VAV3, and PPFIA3 genes are highly methylated in the serum of GC patients compared to healthy ones (all *P* values < 0.01). Altogether, more than 83.3% (40/48) of patients have hypermethylation in at least one of the three analyzed markers in their serum DNA, in contrast to 3 of 25 (12%) healthy controls (*P* < 0.01) (Table 3). The results show that the comethylation reaches 83.3% sensitivity and 88% specificity and therefore may be considered as a panel for the early detection of GC.

### 3.4. Association of Methylation Status in Serum with Clinicopathological Parameters

A summary of the methylation status of the three genes in serum samples and the clinicopathological parameters is shown in Table 4. No significant correlation was found of overall methylation with patients' demographic data, including age, gender, pathological differentiation, depth of tumor invasion, and lymph node metastasis. However, patients with advanced stage disease exhibited a higher serum methylation frequency in VAV3 gene (*P* = 0.019). Interestingly, the methylated OSR2, VAV3, and PPFIA3 in serum exist at the early stage of GC (TNM stages I and II) with a relatively high percentage (OSR2: 59.1%; VAV3: 36.4%; PPFIA3: 45.5%) and therefore could be used to screen GC.

### 3.5. Relationship between Serum Tumor Markers and Serum Methylation

We subsequently compared the diagnostic value between gene methylation and serum tumor markers (Table 5). The sensitivity of combined detection of CEA, CA19-9, and CA-724 reached 33.3%, which was significantly lower than that of MSP assay (33.3% versus 83.3%, *P* < 0.01). Moreover, when we stratified the TNM stages and analyzed the diagnostic value between those two methods in each stage, we found that detection of OSR2, VAV3, and PPFIA3 hypermethylation showed significantly higher sensitivity than that of serum tumor markers (Table 5). Thus, detection of those three genes' methylation in serum was indicated to be more sensitive compared to combined detection of CEA, CA19-9, and CA-724 in the early stage of GC.

## 4. Discussion

In recent years, epigenetic alterations, especially DNA methylation, seem hot to researchers. Studies have shown that aberrant epigenetic modifications occur at the early stage of cancer initiation and play an important role in human carcinogenesis. Therefore, specific methylation biomarkers hold the promise to act as useful tools for early cancer detection. Moreover, those changes could also be detected in the nontarget tissue, especially in blood samples [24, 25]. This gives us a feasibility to detect cancer in a relatively less invasive way.

There have been several studies that previously reported that genes were hypermethylated in GC tissue but were hypomethylated or unmethylated in healthy gastric mucosae [26–30]. However, the majority of studies focused on single gene, which may lead to a relatively low sensitivity in detecting GC cancer. In this study, we evaluated the methylation status of three genes together in both tissue and serum samples to improve the sensitivity. According to Zong et al. [20] research, by using an Infinium HumanMethylation 450 BeadChip array, they identified that OSR2, VAV3, and PPFIA3 are barely methylated in normal cells but highly methylated in GC cells. To further assess the clinical value of methylated OSR2, VAV3, and PPFIA3, we first analyzed those genes' methylation status in GC tissue samples or PCHNTs samples. For each gene (OSR2, VAV3, and PPFIA3), aberrant methylation was significantly more frequent in GC tissue compared to PCHNTs (70.8% versus 4%, 54.2% versus 0%, and 60.4% versus 4%, resp.). We next analyzed serum samples from 48 GC individuals and 25 healthy controls. The result shows that aberrant methylation of those three genes was significantly more frequent in the serum of GC patients compared to healthy subjects (all *P* values < 0.05). The combined sensitivity of at least one positive among the three markers in serum samples reached satisfactory outcome with 83.3% in tumor serum samples (40/48). The analysis between methylation status in the serum and clinicopathological data demonstrated that the methylation status of VAV3 was significantly more frequent in the serum DNA of patients with advanced cancer (TNM stages III and IV) than those with early-stage cancer (TNM stages I and II) (*P* = 0.019). This phenomenon may be caused by the limited sample size, or the percentage of tumor DNA is usually higher in the sera of these advanced GC patients. Finally, our study compared the diagnosis value between promoter hypermethylation and serum tumor markers that are currently used in clinical practice. The result showed that the comethylation of those three genes had a significantly higher sensitivity and specificity than CEA, CA19-9, and CA-724 (83.3% versus 33.3%) (Table 5). Further studies using a greater number of samples needed to be performed to elucidate the diagnostic power of those markers in serum.

For any ideal diagnostic approaches, candidate biomarkers and methods should be with high sensitivity and specificity and relatively noninvasive and could be applied in a cost-effective way. Many researchers use pyrosequencing method to detect DNA methylation to improve accuracy. However, pyrophosphate sequencing could not be widely used in clinical practice for the cost is relatively high and can be a time-consuming operation. In this study, we used conventional methylation-specific PCR (MSP) to detect DNA methylation. MSP has sufficient sensitivity to detect abnormal methylation in a large background of normal DNA [31, 32]. Besides, we detect multiple genes together to improve the sensitivity.

Although epigenetics develops fast in recent years and researchers find that DNA hypermethylation contributes a lot in GC initiation, some obstacles are still needed to be overcome before DNA methylation-based biomarkers can be adopted in clinical practice. The most important one is that guidelines, including procedures, experimental conditions, and instructions, should be standardized to improve the reproducibility of results. Even though many genes have been reported as a biomarker for the early detection, disease monitoring, prognosis, and risk assessment of cancer patients, a lot of valid experiments are still needed to be done before they truly be clinically transformative [33–35].

## 5. Conclusions

In summary, by comparing the comethylation status of OSR2, VAV3, and PPFIA3 in tissue and serum samples between GC patients and healthy individuals, we found that those three genes have a relatively high sensitivity and specificity and therefore may be used as a biomarker for noninvasive screening of GC.

## Figures and Tables

**Table 1:** Methylation-specific primers of OSR2, PPFIA3, and VAV3.

Primer set	Forward primer	Reverse primer	Annealing temperature (°C)	Amp size (bp)
OSR2-M	CGTAGCGCGTGGGATTTTAC	CCAATACTAACAAACCGAAACG	57	100
OSR2-U	GGTTTAGGAGGATGAAGTGT	CACCCTATAACCACCTTTCCCACA	58	85
VAV3-M	GGTTTTTTTTTCGCGCGGGATC	ACGAAAAACGCGCGAAACTCG	57	139
VAV3-U	GGTTTTTTTTTTGTGTGGGATT	CACAAAAAACACACAAAACTCA	57	140
PPFIA3-M	GGTATGTGGTCGTTTGTC	CGAATTACTAATACCGATCTCG	57	98
PPFIA3-U	GGTATGTGGTTGTTTGTT	CAAATTACTAATACCAATCTCA	54	98

**Table 2:** The frequency of promoter hypermethylation of OSR2, VAV3, and PPFIA3 in tissue and serum samples.

	Tissue samples		Serum samples	
	GC	PCHNTs	GC	Normal
OSR2	70.8% (34/48)	4% (1/25)	62.5% (30/48)	8% (2/25)
VAV3	54.2% (26/48)	0% (0/25)	45.8% (22/48)	0% (0/25)
PPFIA3	60.4% (29/48)	4% (1/25)	56.3% (27/48)	4% (1/25)

PCHNTs: paired paracancerous histological normal tissues.

**Table 3:** The positive rate of at least one hypermethylated gene in serum samples.

	OSR2 + VAV3 + PPFIA3 methylation		Positive percent	*P* value
	Positive	Negative		
GC	40	8	83.3%	<0.01^*∗*^
Normal	3	22	12%	

When the markers were used in combination, the test was considered to be positive if one marker reached the threshold and negative if all three markers were negative.

Using chi-square for this statistic.

^*∗*^Statistically significant.

**Table 4:** Correlation between OSR2, VAV3, and PPFIA3 hypermethylation status in serum of GC patients and clinicopathological parameters.

Parameters	Number of cases	OSR2		VAV3		PPFIA3	
		Methylation	*P* value	Methylation	*P* value	Methylation	*P* value
Age							
<60	26	16 (61.5%)	0.881^a^	13 (50.0%)	0.529^a^	14 (53.8%)	0.715^a^
≥60	22	14 (63.6%)		9 (40.9%)		13 (59.1%)	
Gender							
Male	39	23 (59.0%)	0.504^a^	19 (48.7%)	0.186^a^	23 (59.0%)	0.675^a^
Female	9	7 (77.8%)		3 (33.3%)		4 (44.4%)	
Pathological differentiation							
Well + moderate	19	13 (68.4%)	0.493^a^	7 (36.8%)	0.583^a^	11 (57.9%)	0.853^a^
Poor + undifferentiation	29	17 (58.6%)		13 (44.8%)		16 (55.2%)	
Depth of tumor invasion							
Tis, T1a, T1b	5	2 (40.0%)	0.663^a^	2 (40.0%)	0.645^a^	2 (40.0%)	0.694^a^
T2	7	5 (71.4%)		2 (28.6%)		3 (42.9%)	
T3	12	7 (58.3%)		7 (58.3%)		7 (58.3%)	
T4a, T4b	24	16 (66.7%)		11 (45.8%)		15 (62.4%)	
Lymph node metastasis							
N0	16	9 (56.3%)	0.492^a^	5 (31.3%)	0.430^a^	8 (50.0%)	0.514^a^
N1	15	8 (53.3%)		7 (46.7%)		7 (46.7%)	
N2	6	5 (83.3%)		4 (66.7%)		4 (66.7%)	
N3a, N3b	11	8 (72.7%)		6 (54.5%)		8 (72.7%)	
TNM stages							
I and II	22	13 (59.1%)	0.881^a^	8 (36.4%)	0.019^*∗*^	10 (45.5%)	0.165^a^
III and IV	26	17 (65.4%)		14 (53.8%)		17 (65.4%)	

^a^Using chi-square for this statistic.

^*∗*^Statistically significant.

**Table 5:** Sensitivity of serum markers for gastric cancer according to TNM stage.

	TNM stage			
	I (*n* = 9)	II (*n* = 13)	III (*n* = 26)	Total (*n* = 48)
CEA + CA19-9 + CA-724	2 (22.2%)	4 (30.8%)	10 (38.5%)	16 (33.3%)
OSR2 + VAV3 + PPFIA3 methylation	7 (77.8%)	10 (76.9%)	23 (88.5%)	40 (83.3%)
